# DVNE-DRL: dynamic virtual network embedding algorithm based on deep reinforcement learning

**DOI:** 10.1038/s41598-023-47195-5

**Published:** 2023-11-13

**Authors:** Xiancui Xiao

**Affiliations:** 1 School of Information Engineering, Shandong Management University, Ji’nan, 250357 China; 2 Key Laboratory of TCM Data Cloud Service in Universities of Shandong, Ji’nan, China

**Keywords:** Energy infrastructure, Information technology

## Abstract

Virtual network embedding (VNE), as the key challenge of network resource management technology, lies in the contradiction between online embedding decision and pursuing long-term average revenue goals. Most of the previous work ignored the dynamics in Virtual Network (VN) modeling, or could not automatically detect the complex and time-varying network state to provide a reasonable network embedding scheme. In view of this, we model a network embedding framework where the topology and resource allocation change dynamically with the number of network users and workload, and then introduce a deep reinforcement learning method to solve the VNE problem. Further, a dynamic virtual network embedding algorithm based on Deep Reinforcement Learning (DRL), named DVNE-DRL, is proposed. In DVNE-DRL, VNE is modeled as a Markov Decision Process (MDP), and then deep learning is introduced to perceive the current network state through historical data and embedded knowledge, while utilizing reinforcement learning decision-making capabilities to implement the network embedding process. In addition, we improve the method of feature extraction and matrix optimization, and consider the characteristics of virtual network and physical network together to alleviate the problem of redundancy and slow convergence. The simulation results show that compared with the existing advanced algorithms, the acceptance rate and average revenue of DVNE-DRL are increased by about 25% and 35%, respectively.

## Introduction

In modern society, with the increasing number of users and various types of network services, the current Internet architecture and management methods are facing great pressure, so many promising network management technologies emerge. Network virtualization, as one of the key technologies of the next generation Internet, aims to overcome the resistance caused by the current Internet's response to changes in network structure^1–4^. Network virtualization can decouple network services from their physical network hardware, and allow network users to program their own network services. Through network virtualization technology, traditional network providers can be divided into two roles: service providers and physical network providers. The former's task is to create customized virtual networks (VN_S_) to provide end-to-end network services to users, while the latter mainly maintains infrastructure provider (InP) devices so that service providers can adopt them to host their services at an acceptable business cost^5,6^. It turns out that good coordination between these two roles can improve the practicality and commercial value of physical network devices. The application of network virtualization technology depends on the algorithms that can instantiate the virtual network on the infrastructure to optimize the layout of service-related indicators. This algorithm is often referred to as "virtual network embedding (VNE)"/"virtual network mapping (VNM)" algorithm. VNE solves how to provide virtual network request (VNR) and allocate physical network nodes and link resources for them reasonably and efficiently. Virtual network requests (VNRs) usually contain multiple constraints such as node attributes and link attributes (CPU, storage, bandwidth, etc.), request admission, and dynamic requirements. Therefore, if all the above network constraints are taken into account, the VNE problem is going to be a very difficult problem. It has been proved that the solution process of VNE is also NP-hard even if only node and link constraints are considered, which means that accurate solutions of VNE problems cannot be obtained in the actual complex network environment^7,8^.

At present, in order to reduce computational complexity and running time, virtual network embedding problems usually add assumptions or simplify constraints in a heuristic way to reduce solution space and obtain suboptimal solutions within acceptable complexity. However, traditional virtual network solutions often have the following drawbacks:

1. Neglecting the dynamic changes of users in the process of network service business, including changes in the number of user resource requirements and network topology corresponding to business types.
2. Manually develop a set of rules and assumptions. Although the model and constraint rules are simplified, it is often not possible to automatically detect complex and time-varying network states to provide the most reasonable network embedding scheme for the current network state.

**Figure 1:**
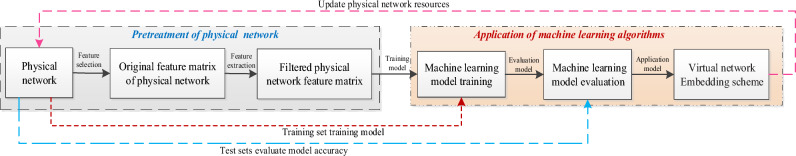
VNE based on machine learning algorithm.

In recent years, machine learning algorithms have made exciting new achievements in the fields of big data, machine learning, and artificial intelligence, especially in natural language understanding and object detection. Machine learning algorithms can collect a large amount of data within a period of time for processing, and automatically learn statistical information from the data, so as to make classification or prediction^9–11^. In particular, deep reinforcement learning, as a widely used algorithm in machine learning, integrates the powerful understanding ability of deep learning in perceptual problems and the decision-making ability of reinforcement learning, demonstrating great potential in handling complex tasks (such as Go^12^) or complex control tasks (such as autonomous driving^13^). For the virtual network embedding problem in this article, as shown in Fig. 1, virtual network requests arrive at different time points, and a large amount of historical data and embedded knowledge are generated over time. Specifically, the handling of new virtual request events of the same type is similar, as they are calculated and allocated a certain amount of resources based on the requirements of network users. For network embedding scenarios, it is envisioned to introduce deep learning to perceive and understand current network information through historical data and embedded knowledge, while utilizing reinforcement learning decision-making ability to select appropriate embedding methods for new VNRs, which will effectively solve VNE problems. Unfortunately, existing research often fails to pay attention to the historical data and embedded knowledge mentioned above, and in fact, utilizing these historical data and embedded knowledge to assist in the subsequent virtual network embedding process is very meaningful work.

In view of the above analysis, this article introduces deep reinforcement learning into the virtual network embedding problem in order to dynamically optimize the virtual network mapping process. We focus on deep reinforcement learning methods in machine learning and discuss how reinforcement learning agents (RLA) can be used to detect real-time network state and enable dynamic virtual network mapping. We first divide the network request data into a training set and a test set for training a deep reinforcement learning agent and evaluating its performance, respectively. Then, an artificial neural network called policy network is designed to observe the state of the physical network and output the node mapping results, while using policy gradients to train the historical network request data through backpropagation. The exploration strategy is used in the training phase to find a better solution, while the greedy strategy is used in the evaluation phase to fully evaluate the effectiveness of the agent. Finally, this article proposes a dynamic virtual network embedding/mapping algorithm DVNE-DRL based on reinforcement learning, which can use historical network request data to obtain mapping knowledge and apply it to deep reinforcement learning based on policy network, so as to efficiently solve the virtual network embedding problem in this article. In summary, the main work of this article is as follows:

1. We propose a new dynamic network embedding framework, in which the virtual network topology and resource requirements change dynamically with the workload and connection form in different time periods, which can better meet the more actual network state of network users.
2. We analyze the feature relationship between virtual network and physical network, extract effective network feature information as deep reinforcement learning training sample data, reduce RLA training redundancy and improve convergence speed.
3. We establish a DRL model based on solving VNE problems, and focus on designing corresponding reward and punishment functions and information feedback mechanisms to improve mapping efficiency and reduce the average cost of network embedding.
4. We design and implement a dynamic virtualization algorithm based on deep reinforcement learning strategy network (DVNE-DRL), and test the performance of the algorithm by comparing with the three algorithms through simulation experiments.

Simulation results show that RLA can extract knowledge from historical data and extend it to incoming requests. In addition, by strengthening the interaction between the learning agent and the environment, and using the reward and punishment mechanism to make itself iterative learning, the virtual network embedding process is automatically optimized. The proposed DVNE-DRL algorithm is significantly superior to superior to some existing new virtual network mapping algorithms in terms of long-term average network revenue and acceptance rate.

The remainder of this article is structured as follows: Section "Literature review" discusses the literature that is related; Section "Network model and problem description" provides network model and problem description; and Section "Algorithm design and implementation" discusses the proposed algorithm design and implementation. The proposed algorithm experimental results and data are discussed in Section "Evaluation index and result evaluation", along with a case study, and Section "Conclusions and future work" brings the work to an end by summarizing the content and providing a proposal for further extension.

## Literature review

### Traditional VNE algorithm

The VNE problem mentioned above is a typical NP-hard problem, which means that only an approximate optimal solution can be obtained through the correlation design algorithm^7,8^. In view of this, many researchers have designed different styles of embedding scenes, and thus proposed corresponding VNE algorithms in recent years. There are many different division rules for the classification of virtual network embedding algorithms. For example, according to the accuracy of the embedding result, it can be divided into exact solution algorithms and heuristic/meta-heuristic algorithms. Among them, the exact solution algorithm can find the optimal solution within a certain time range. When the scale of the problem is large, the application of the exact solution algorithm usually has two purposes: provide a feasible solution to the problems and to provide an initial solution for the heuristic method. For example, Liu^14^ et al. reduced the process of node embedding and link embedding to a discrete nonlinear combinatorial optimization problem, proposed a new exact deembedding method, and established a mathematical description of the relationship between node embedding and link embedding. Most of the heuristic/meta-heuristic algorithm literature focuses on the study of related natural body algorithms. For example, Song et al.^16^ designed a single-stage dual heuristic particleswarm optimization algorithm to solve VNE, aiming at coordinating node embedding and link embedding. Heuristics play an active role in certain social applications and technological contexts. However, the analysis shows that traditional VNE solutions have several similar shortcomings. More importantly, the dynamic characteristic of VNE is often ignored during embedding, such as resource allocation of virtual nodes and links, virtual topology, number of physical network resources, and network embedding process.

### Dynamic VNE algorithm

According to whether the user resource requirements change dynamically in the embedding process, it can be divided into static virtual network embedding algorithm and dynamic virtual network embedding algorithm. Dynamic VNEs have higher complexity and uncertainty than static VNEs. Generally, the dynamism of a virtual network includes the following points: the dynamism of the underlying physical network, the dynamism of virtual network requests, and the dynamism of the arrival and departure of virtual network events during the network service process. Thiruvenkadam^17^ et al. proposed a fuzzy based multi-criteria decision making technique, using membership function of node parameters to prepare node embeddings, and verifies the proposed strategy under dynamic physical infrastructure conditions. Minardi^18^ et al. proposed a hybrid binary linear programming (MBLP) formula for the dynamic topology-aware VNE (DTA-VNE) algorithm, in which dynamic embedding is planned for each virtual network request (VNR) given prior information about the evolution of the network over time. Xiao et al.^19^ proposed a dynamic resource requirement prediction algorithm based on RBF incremental design, which uses the improved RBF to predict user resource demand and real distributes resources according to the predicted results to reduce resource allocation redundancy. Thakur et al.^20^ established a multi-domain SDN network VNE model based on irregular cell learning automata (ICLA), extended VDN-CLA by considering dynamic traffic migration, and realized resource optimal routing to improve throughput and reduce end-to-end latency. He et al.^21^ used spectral clustering to extract physical network features to distinguish the dynamic regions of interest and find embedding regions with energy-saving potential of virtual networks.

### VNE algorithm based on machine learning

In most previous research work, physical network information and virtual network embedding knowledge hidden in historical network request data are often ignored. However, in practice, the historical virtual network requests can reflect the time distribution and resource demand characteristics of the future network. In recent years, some ideas of machine learning algorithms have been introduced into the design of virtual network embedding algorithms, and good results have been achieved^22–24^. Practice has proven that RL has superior decision-making ability due to the effective interaction between learning agents and the environment. Generally, the machine learning algorithm follows the overall design of a time line, in which at each time step t, the agent interacts with the environment to obtain a new environment state st, then takes corresponding candidate actions according to the environment state, and finally transfers the environment state to the lower st + 1. At present, in solving VNE problems with machine learning, Lim et al.^25^ proposed a layered cooperative multi-agent reinforcement learning algorithm to optimize the VNE problem by maximizing the average benefit, minimizing the average cost, and increasing the request acceptance rate. Yuan et al.^26^ proposed a VNE algorithm based on RL model and found the corresponding VNE embedding method through RL proxy. Yao et al.^27^ proposed a VNE algorithm based on single-layer policy network. The RL agent collects the historical embedding data of the virtual network and optimizes the VNE embedding strategy using the policy gradient descent method. The VNE algorithm proposed by Yao et al.^28^ and Dolati et al.^29^ combines deep learning (DL) and RL, which also provides some inspirations for solving VNE problems. At present, VNE solutions based on machine learning mainly adopt DL, RL or graph theory^30–32^. Among, RL can connect and map environments and behaviors to maximize the functional value of intelligent systems. In RL agent learning, the environment evaluates the quality of the current action through feedback rather than directly determining which action the reinforcement learning system (RLS) should take. In practical applications, the external environment is usually complex and difficult to form significant information characteristics, that is, the RL agent provides little information, which also leads to the need for RLS to learn from their own experience to complete the target optimization. In view of the above mentioned, the VNE problem in this article also has the similar problems mentioned above, such as the complex and changeable network environment and insufficient information. Therefore, deep reinforcement learning is gradually beginning to play an important role^33^ and has become a popular mainstream method to solve the VNE problem.

## Network model and problem description

### Network model

**Figure 2:**
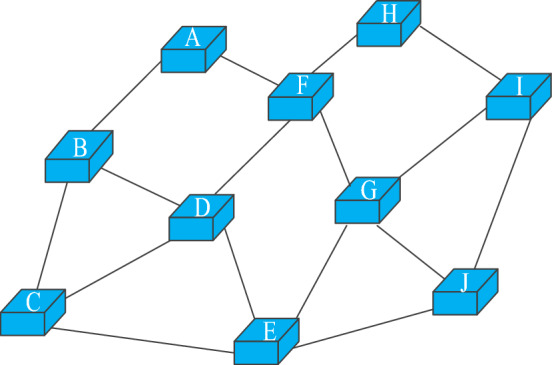
Physical network example.

**Figure 3:**
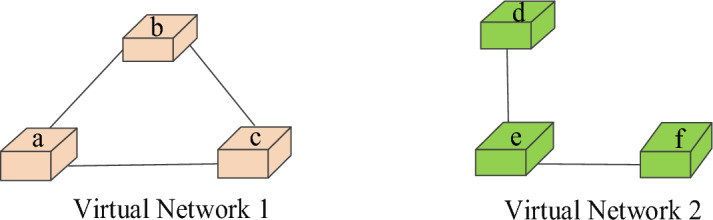
Two virtual network examples.

Virtual network embedding is the process of allocating corresponding physical resources to logical networks with resource requirements on shared physical networks. The shared physical network can be abstracted as a weighted undirected graph, marked as $G^{s} = (N^{s} ,L^{s} ,A_{N}^{s} ,A_{L}^{s} )$. Figure 2 shows a physical network topology with more nodes and links. The network constructed according to the user's virtual network request, namely virtual network, is usually marked as undirected weighted graph. A VNR can also be modelled as $G^{v} = (N^{v} ,L^{v} ,A_{N}^{v} ,A_{L}^{v} )$. Figure 3 shows two virtual networks with different topologies. Table 1 describes the network parameters and their meanings.

**Table 1:** Network parameters and meaning.

Parameters	Meaning
$$n^{s} $$	Physical network node
$l^{s}$	Physical network link
$$N^{s} $$	Node set of physical network
$L^{s}$	Link set of physical network
$A_{N}^{s}$	Node attributes of the physical network
$A_{L}^{s}$	Link attributes of the physical network
$n^{v }$	Virtual network node
$l^{v}$	Virtual network link
$N^{{\text{v}}}$	Node set of virtual network
$L^{v}$	Link set of virtual network
$A_{N}^{{\text{v}}}$	Node attributes of virtual network
$A_{L}^{v}$	Link attributes of virtual network

### VNE model and description

**Figure 4:**
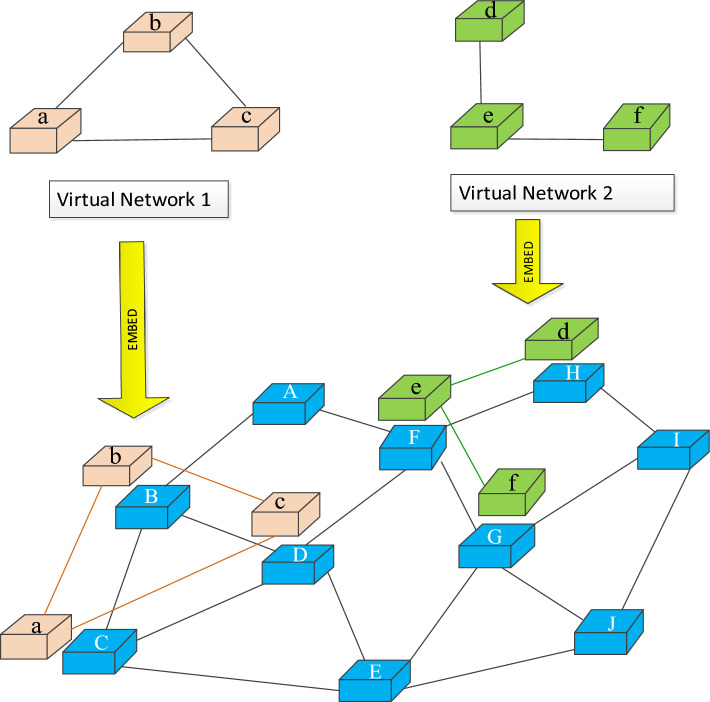
Embedding of two VNs.

As in the previous study, the VNR embedding process is divided into node embedding and link embedding stages. Since VNE is a NP-hard problem, according to different computing models and embedding strategies, various available embedding schemes can be generated to meet the virtual request network generated by Internet users. Figure 4 shows an example of two virtual network request embedding schemes.

As shown in Fig. 4, VNR nodes/links are mapped to different physical nodes/links, and these physical nodes/links can meet their multiple resource and geographical location constraints at the same time. The embedding scheme shown in Fig. 4 can be represented as node_embedding = {a->B, b->C, c->D} and link_ embedding = {ab->BC, bc->CD, ac->BD} of VN1, and node_ embedding = {d->H, e->F, f->G} and link_ embedding = {de->HF, ef->FG} of VN2.

### Dynamic network embedding

We assume that VNRs arrive successively on the time axis. Whenever a new VNR arrives, the network provider will decide to accept or reject it according to the relevant attribute constraints. In reality, network providers usually maximize their revenue and benefits by effectively utilizing limited physical resources. In view of this, we define network revenue as:

1 $$ \Re \left( {G_{i}^{v} } \right) = T_{i}^{v} \times \left[ {\omega_{1} \sum\limits_{{n^{v} \in N^{v} }} {P(n^{v} ) + \omega_{2} \sum\limits_{{l^{v} \in L^{v} }} {B(l^{v} )} } } \right] $$

where, $T_{i}^{v}$ represents the life cycle of the virtual request $G_{i}^{v}$, $P(n^{v} )$ represents the computing resources, $B(l^{v} )$ represents link, and $\omega_{1}$, $\omega_{2}$ are weight parameters.

**Figure 5:**
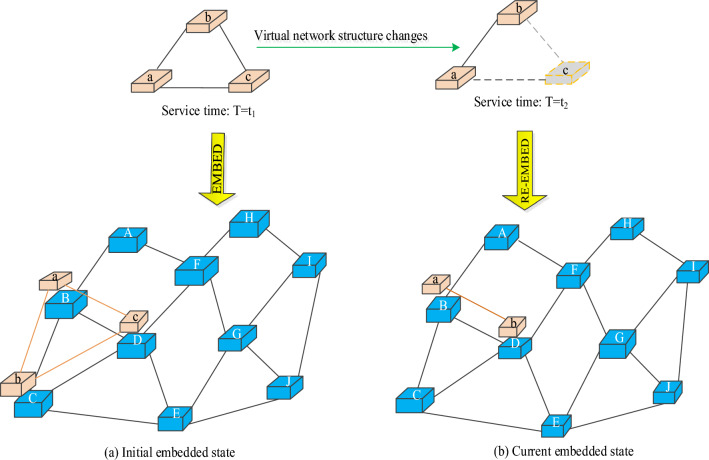
Dynamic Network Embedding example.

Generally, changes in the number of network users will lead to changes in the topology of the virtual network, and fluctuations in business workload will affect the configuration of virtual network resources. The virtual network whose structure configuration or resource configuration changes with time is called dynamic virtual network, and the embedding process is called dynamic VNE. As shown in Fig. 5, a dynamic virtual network embedding scenario needs to adjust the embedding scheme within the network service time to maximize revenue.

**Figure 6:**
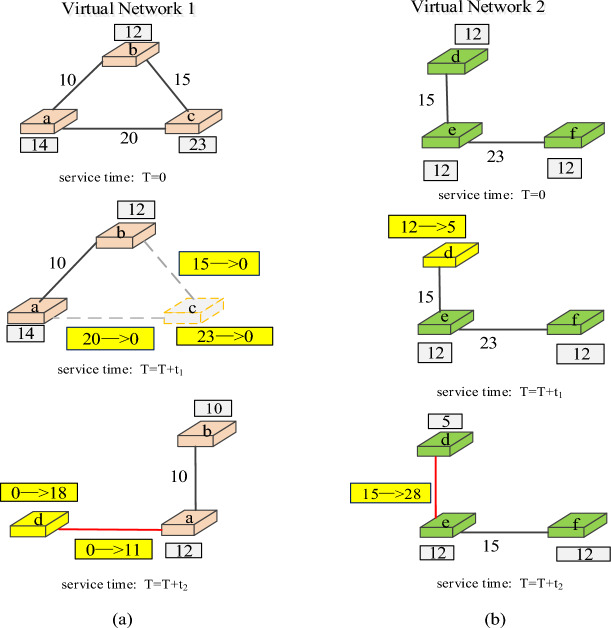
Dynamic virtual network. (**a**) Network structure changes due to the number of users. (**b**) Network resource allocation changes due to user workload.

Figure 6 shows the change process of the structure configuration and resource configuration of a dynamic virtual network, and T represents different continuous time periods in the VNE process. It is worth noting that such dynamic changes in the number of resources or topology are equivalent to adding more VNR event nodes at the time point of event change. Therefore, in the subsequent design of DVNE-DRL algorithm, the historical embedding data is actually extended to a large extent, which is conducive to training RAL and improving its performance.

## Algorithm design and implementation

DRL combines the advantages of deep learning and reinforcement learning, in which the agent can be well trained in the policy network to obtain better dynamic VNE results. We will introduce the embedding principle and process of DVNE-DRL algorithm, including the network feature extraction, DRL policy network, reward function for VNE, and pseudo-code description of DVNE-DRL algorithm.

### Markov decision processes

**Figure 7:**
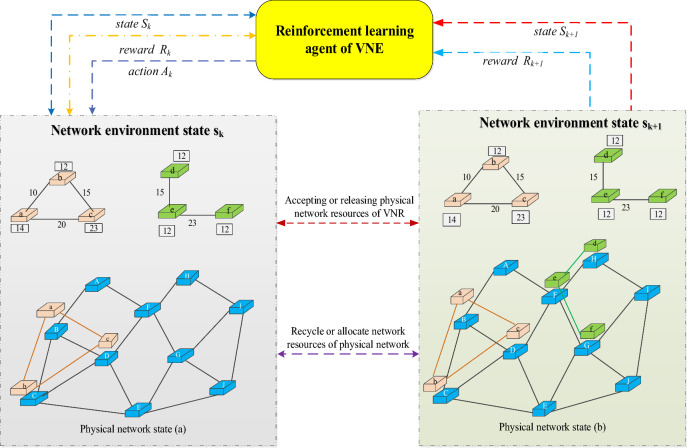
VNE process with RLA interpretation.

The virtual network embedding problem is actually to find a physical network that meets the node and link constraints for each incoming virtual network request. We can model the network embedding process as a Markov decision process, and then use reinforcement learning to solve it. The specific description process is shown in Fig. 7.

In general, a Markov decision process can be defined as a quintuple $\left( {S,A,R,P,\gamma } \right)$ where $s$ is the state space, $A$ is the action space, and $R:s \times a \to R$ is the pair of actions a reward, $P:s \times a \times s \to [0,1]$ is a state transition distribution, that is, the probability of selecting action a to transition to state s in state s' can be expressed as formula (2):

2 $$ P\left( {s^{\prime},a,s} \right) = Pre(s_{t + 1} = s^{\prime}|a_{t} = a,s_{t} = s) $$

Accordingly, we can model the virtual network embedding/mapping process as a series of decision instances. The agent receives virtual network requests, solves the virtual node embedding process for it, and harvests corresponding rewards. The agent's goal is to maximize this reward R. We assume that a physical network has N nodes and M links, and at a certain point in time there is a VNR containing X virtual nodes and Y virtual links, denoted as $G^{v}$, as we define a virtual network corresponding to a finite time, the embedding process is a Markov decision process, which is a $M{\text{ark\_G}}^{{\text{v}}}$ decision process. The reinforcement learning decision agent will continuously select x physical nodes for it according to the features of the virtual network to map the node request in the virtual network request. At this time, the x decision instance is generated. If all nodes are successfully mapped, the $M{\text{ark\_G}}^{{\text{v}}}$ decision process will reach a temporary state at the next moment. We assume that in a given stat s', the agent assigns a physical node ns to a virtual node n^v^. Then the state and state space can be expressed as formula (3) and formula (4) respectively:

3 $$ S_{t} = \left( {N_{t}^{v} = N_{t - 1}^{v} \backslash \left\{ {n_{t - 1}^{v} } \right\},N_{t}^{s} = N_{t - 1}^{s} \backslash \left\{ {n_{t - 1}^{s} } \right\}} \right) $$

4 $$ A_{t}^{V} = \left( {n_{t}^{V} ,n^{s} } \right):\forall n^{s} \in \left\{ {N_{t}^{s} \cap N^{s} \left( {n_{t}^{V} } \right)} \right\} \cup \left\{ \chi \right\} $$

Among, the reinforcement learning agent needs to select a node from the action space defined in formula (4),$\chi$ represents the result that the force is converted into arbitrary action, and the $n^{s}$ is used as the embedding node of the virtual node, which strengthens the reward obtained by the agent accounting. How to extract network node features as the basis for selecting embedding nodes will be described in Section "Network feature extraction".

### Network feature extraction

DVNE-DRL in this article combines deep learning and reinforcement learning algorithms, so feature extraction is very important for accurately measuring the embedding between virtual nodes and physical networks. Since sufficient network features can better train network agents and improve embedding accuracy, we extract features from two (virtual nodes and physical nodes) to form a feature matrix for environmental agent training. A deep understanding of the feature relationship between the two is essential to fundamentally understand the reinforcement learning agent to effectively map its state. In a real network embedding environment, physical nodes and virtual nodes have many characteristics, such as CPU resources, location, connection bandwidth and degree. There are also many feature points used to describe the relationship between them. We have extracted the following five features:

(1) CPU computing power gap (GP_CPU)

The CPU of a physical node is the most important feature of a embedding node. If the computing resources of the physical node are sufficient, the node resource request in VNR will be mapped to the physical node with high probability. When the physical node has less computing resources, the virtual node will give priority to other physical nodes with sufficient computing resources, so as to avoid bottleneck nodes and improve the load balancing performance of the entire network. Therefore, the CPU computing power gap between virtual nodes and physical nodes is the most important and decisive feature.

5 $$ GP\_CPU\left( {n_{k}^{s} } \right) = CPU(n_{k}^{s} ) - CPU(n_{i}^{v} )\mathop {}\limits_{{}}^{{}} $$

(2) Distance between nodes

In the process of virtual node embedding, selecting nodes with reasonable geographical locations for embedding can greatly reduce the number of forwarding nodes in link embedding, thereby reducing link bandwidth occupancy and utilizing network resources more reasonably. Therefore, the distance feature extraction between nodes is an important factor to measure the success rate of embedding.

6 $$ D{\text{S}}\_NN\left( {n_{k}^{s} } \right) = \sqrt {(x_{{n_{k}^{s} }} - x_{{n_{i}^{v} }} )^{2} + (y_{{n_{k}^{s} }} - y_{{n_{i}^{v} }} )^{2} } $$

(3) Degree gap (DG_NN)

The degree of a physical node represents the degree to which the physical node is connected to other physical nodes. Nodes with better connectivity will be preferentially selected as embedding nodes, which is beneficial to improve the success rate of subsequent link embedding and the network acceptance rate on the one hand. On the other hand, it can effectively prevent the occurrence of blocking in the physical network.

7 $$ DG\_NN\left( {n_{k}^{s} } \right) = Degree(n_{k}^{s} ) - Degree(n_{i}^{v} ) $$

(4) Sum of bandwidth gap (GP_BW)

The bandwidth connected to the physical nodes and the connectivity between the nodes are described from the perspective of bandwidth. The richer the bandwidth resources, the greater the connection probability between the virtual node and the physical node, that is, the more ideal the link embedding option.

8 $$ GP\_BW\left( {n_{k}^{s} } \right) = \sum\limits_{{l_{s} \in L(n_{k}^{s} )}} {bw} (n_{k}^{s} ) - \sum\limits_{{l_{v} \in L(n_{i}^{v} )}} {bw} (n_{i}^{v} ) $$

(5) Sum of the distance between the mapped node and the node (DS_MNN)

The sum of the distances between the mapped node and the node is the sum of the distances from other nodes in the virtual network request to the node before the node request is mapped. This attribute is zero when the first node in the request is mapped. When the second node is mapped, the attribute is the distance from the mapped node to this node. Similarly, when the next request node is mapped, the attribute is the distance from all other nodes that have been mapped to this node. This attribute describes the total distance between the mapped nodes, so the smaller the attribute is, the better the node should be selected for the embedding request. That is, a combination of nodes with a smaller number of hops between nodes can effectively save link resources of the physical network. In other words, extracting the feature of the sum of the distance between the virtual node that has been mapped on the physical node and the current virtual node is very important for reducing the embedding cost.

9 $$ DS\_MNN\left( {n_{k}^{s} } \right) = \sum\limits_{{all(n_{m}^{v} \uparrow n_{k}^{s} )}} {\sqrt {(x_{{n_{i}^{v} }} - x_{{n_{m}^{v} }} )^{2} + (y_{{n_{i}^{v} }} - y_{{n_{m}^{v} }} )^{2} } } $$

In fact, physical nodes have many more characteristics than those listed above. The physical network information increases as the features increase, so the learning agent is more capable. But extracting too many features increases the computational complexity. Use the extracted five node features to describe the node state, and then normalize the feature values to 0–1 to remove the peculiar sample data to get X'.

10 $$ X^{\prime} = \frac{{X - X_{\min } }}{{X_{\max } { - }X_{\min } }} $$

Let S_k_ denote the feature vector of the k^th^ physical node. All the features are spliced together, and the attribute matrix *A* of the node can be obtained. We train the learning agent using the feature vectors as input to the policy network.

11 $$ A_{k} = \left( {GP\_CPU\left( {n_{k}^{s} } \right),D{\text{S}}\_NN\left( {n_{k}^{s} } \right),DG\_NN\left( {n_{k}^{s} } \right),GP\_BW\left( {n_{k}^{s} } \right),DS\_MNN\left( {n_{k}^{s} } \right)} \right)^{T} $$

All the above physical nodes A_k_ are constructed into a feature matrix for strengthening learning input.

12 $$ M_{f} = \left( {v_{1} ,v_{2} ,v_{3} ....v_{n} } \right)^{T} $$

### DRL policy network

**Figure 8:**
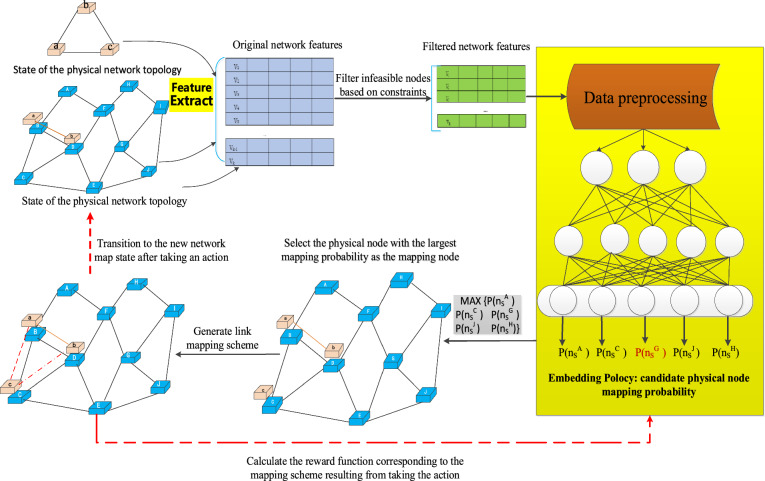
RL framework of a VNE problem.

The state space of the VNE problem is a continuous value, so this article constructs a node embedding policy network, introduces a policy-based method in reinforcement learning, and uses an agent to optimize the entire training model. The policy network includes input layer, convolutional layer, softmax layer, filter layer and output layer as shown in Fig. 8. The physical node selected for the virtual node to be mapped using the policy network.

In the input layer, the feature matrix of the physical network nodes is calculated and passed to the convolutional layer, the matrices are convolved in the convolutional layer, resulting in a vector representing the available resources for each physical node.

13 $$ h_{k} = \left\{ {\begin{array}{*{20}c} {a \cdot V_{k} + b} & {if\,a \cdot V_{k} + b > 0\,} \\ 0 & {otherwise} \\ \end{array} } \right. $$

*h* represents the kth output of the softmax, *a* is the weight vector, and *b* is the bias term. In the softmax layer, *k* is converted into the probability of each physical node being selected, and a high probability node is selected for embedding to obtain a better embedding result. The formula (14) for calculating the probability *P*_*k*_ is as follows.

14 $$ p_{k} = \frac{{e^{{h_{k} }} }}{{\sum\limits_{i} {e^{{h_{i} }} } }} $$

For those nodes that do not meet the basic rules of embedding, they are filtered in the filtering layer, and the nodes with sufficient computing resources are filtered out. And recalculate the probability distribution at the output layer, and the input results are as follows.

15 $$ p = (p_{1,} p_{3,} p_{5,} ...p_{k} ) $$

In this article, we use the gradient strategy method to train the node embedding strategy network in the multi domain network. A handmade label is introduced into the policy network to temporarily consider whether each decision made by the reinforcement learning agent is correct. Suppose that the node in the multi domain physical network is selected, then the handmade label in the policy network will be a vector. Except the first label is 1, all other labels will be 0. The cross entropy loss function is shown in formula (16).

16 $$ L\left( {\text{y,p}} \right){ = - }\sum\nolimits_{{\text{k}}} {\left( {{\text{y}}_{{\text{k}}} log{\text{(p}}_{{\text{k}}} {)}} \right)} $$

where, $y_{k}$ represents the k^th^ node of the manual label and $p_{k}$ represents the kth output of the policy network. At the same time, the small batch gradient descent method is used to dynamically update the parameters of the strategy network. In the iteration, *batch_size* samples were selected to complete an update, and parameters were introduced to adjust the size of the gradient and the training calculation speed.

### Reward functions for VNE

We know that reinforcement learning uses an unsupervised way, there are no labels in the training set, and only the reward of the agent is used to judge whether the model is used or not. If the reward is larger, the current action is considered valid, and if the reward is smaller or negative, the action is considered invalid and needs to be corrected in time.

In the virtual network embedding problem, if the virtual network request is successfully mapped, it will return a positive reward, otherwise, it will return a negative reward. However, the significance of whether an action is successful or not may be different, because it may lead to many possible states and make the return after long-term accumulation different. Generally, the process of designing reward functions that meet the expected goals more accurately in order to distinguish the degree of good behavior is called reward shaping. In VNE, the learning agent takes action to ensure that the embedding is successful. In order to improve the network acceptance rate, we should first encourage successful embedding actions. For operations that meet the requirements of virtual resources, we set the reward to 1, otherwise it is 0.

17 $$ AR(G^{v} ,t) = \left\{ {\begin{array}{*{20}c} {\lambda_{AR} } & {G^{v} is successfully\,\,embeded} \\ { - \lambda_{AR} } & {otherwise} \\ \end{array} } \right. $$

Second, the actions taken by the learning agent are to be maximally cost-effective. That is, when dealing with the same VNR, a better embedding row action will consume less network resources (physical link resources and physical node resources), and the network revenue and cost function are respectively defined as the following formulas (10) and (11), so we add another factor to the reward function:

18 $$ Revenue(G^{v} ,t){ = }\sum\limits_{{n^{v} \in N^{v} }} {cpu(n^{v} )} + \sum\limits_{{l^{v} \in L^{v} }} {bw(l^{v} )} $$

19 $$ Cost(G^{v} ,t){ = }\sum\limits_{{n^{v} \in N^{v} }} {cpu(n^{v} )} + \sum\limits_{{l^{v} \in L^{v} }} {bw(l^{v} ) \times hops(l^{v} )} $$

20 $$ RC(G^{v} ,t) = \frac{{Revenue(G^{v} ,t)}}{{Cost(G^{v} ,t)}} $$

Network load balancing can reduce the occurrence of bottleneck nodes, thereby increasing the network acceptance rate. To balance the workload among the physical network nodes, learning agents are encouraged to choose physical nodes with more node resources. The selection of the node embedding scheme directly affects the later link embedding. Link load balancing is closely related to the user’s information transfer efficiency. In order to avoid link congestion, a link with rich resources should also be selected for embedding. Therefore, we use the above two links. Influencing factors are added to the reward function:

21 $$ LB(G^{s} ,t) = \alpha \frac{{re\_cpu_{t} (n^{s} )}}{{ini\_cpu_{t} (n^{s} )}} + \beta \frac{{re\_bw_{t} (l^{s} )}}{{ini\_bw_{t} (l^{s} )}} $$

where $\alpha ,\beta$ are the balancing parameters of node load balancing and link load balancing, which affect the reward function to the same extent.

During the training process, the policy may fall into a sub-optimal state, so we should avoid repeatedly generating the same action embedding policy, and encourage the policy to transfer to other actions that are not selected. Therefore, we want to set an eligibility check at a certain period to ensure that the current training does not get stuck in repeated steps. To this end, this factor should also be considered in the reward function design:

22 $$ EC_{t} (m) = \left\{ {\begin{array}{*{20}c} {ck(EC_{t - 1} [m] + 1)} & {m = = \alpha_{t} } \\ {ck \times EC_{t - 1} [m]} & {m \ne \alpha_{t} } \\ \end{array} } \right. $$

where *c*_*k*_ is the decay factor, which shrinks a little at each time step, which ensures that frequently selected actions can be kept for a period of time, while unselected actions will gradually decay to 0. The above approach filters out actions that are not frequently chosen, thereby eliminating the danger of training falling into a suboptimal state.

In summary, the $\alpha_{t}$ action reward function can be defined as follows:

23 $$ Reward\left[ {G^{v} ,G^{s} ,a_{t} } \right] = \frac{{AR(G^{v} ,t) \times RC(G^{v} ,t) \times LB(G^{s} ,n_{k} )}}{{EC_{t} (a_{t} ) + \mu }} $$

### DVNE-DRL algorithm implementation

DVNE-DRL algorithm uses the trained policy network to complete the entire embedding process dynamically and outputs the global optimal virtual network embedding scheme. Compared to a custom set of rules and assumptions for manual rules, simple mathematical calculations lead to embedding schemes. DVNE-DRL interacts with the environment and uses the reward learning optimal embedding mechanism for dynamic feedback, which can effectively discover the representation of the physical network and the virtual network. The relationship between requests is detected, and the dynamic changes of the virtual network over time are detected to efficiently complete the virtual network embedding. Algorithm I and algorithm dichotomy show the training and testing process of the network. Algorithm III shows the overall processing process of deep reinforcement learning in online network embedding. The specific pseudocode is as follows:

**Algorithm I:**
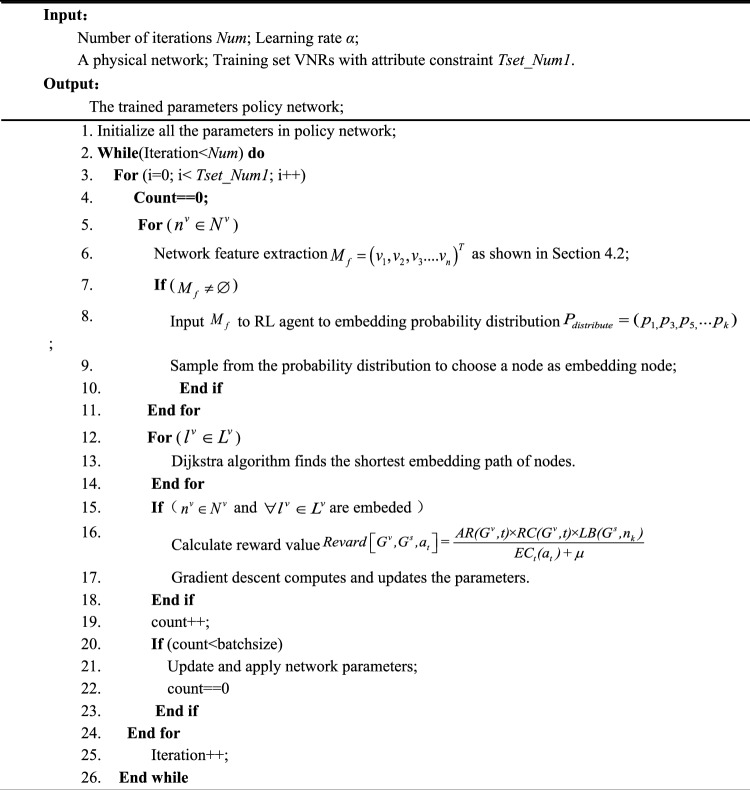
Network training process

**Algorithm II:**
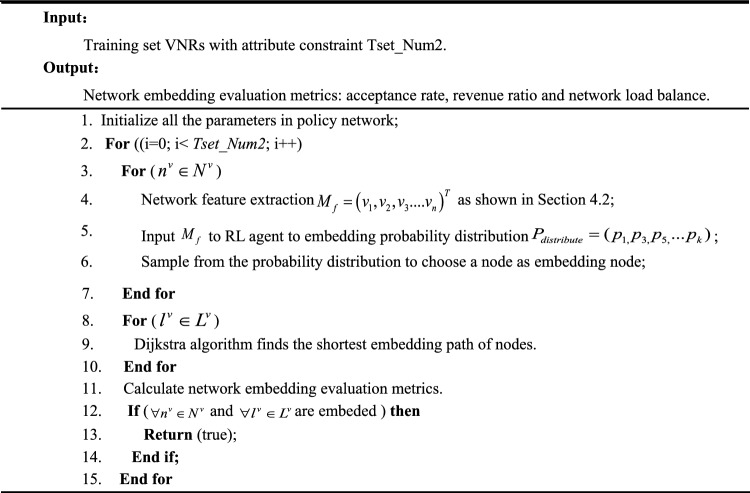
Network test process

**Algorithm III:**
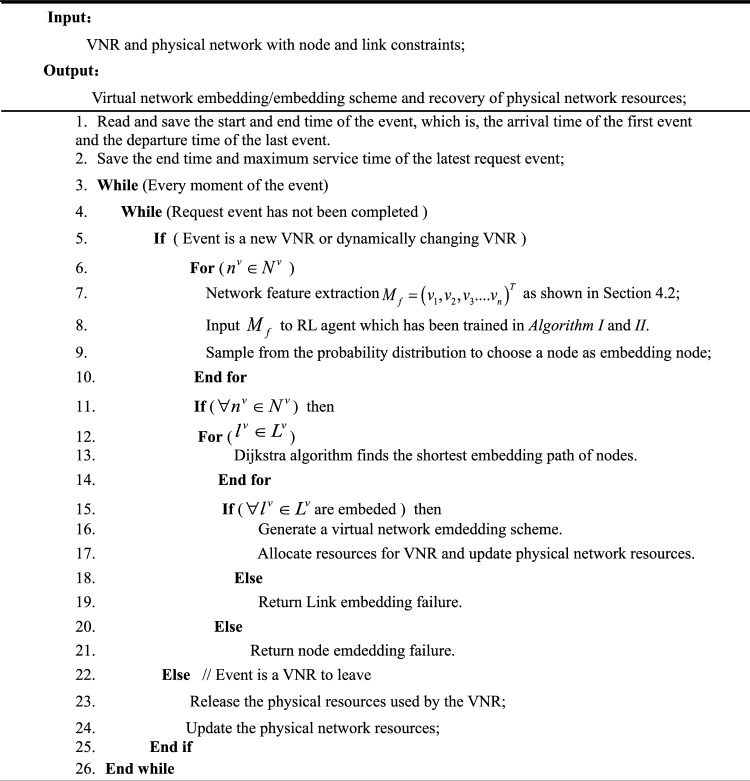
DVNE-DRL network embedding algorithm

### Complexity analysis

In this section, we analyze the time complexity of the DVNE-DRL algorithm. We know that the computational complexity of a deep neural network algorithm is defined as the number of operations of the model. The number of operations of a deep neural network with fully connected layers is determined by the number of computing units of the network model, including the input dimension, the number of neurons in each hidden layer, the output layer dimension, and the number of layers of the neural network. In the VNE scenario of this paper, assuming that the dimension of the input adjacency matrix is $n$ and the dimension of the attribute matrix embedded is $m$, the time complexity of the network input can be roughly expressed as $O\left( {n^{2} m} \right)$. For the DRL, the actor network and the critic network should be considered at the same time, and the number of network layers of both are represented by $\chi^{a}$ and $\chi^{c}$ respectively, while the number of neurons of the corresponding $\chi$ layer can be represented by $net_{\chi }^{a}$ and $net_{\chi }^{c}$. According to the above analysis, the time complexity of algorithm DVNE-DRL can be expressed as $O\left( {n^{2} m + \sum\limits_{\chi = 0}^{{\chi^{a} { - }1}} {net_{\chi }^{a} } net_{\chi + 1}^{a} + \sum\limits_{\chi = 0}^{{\chi^{c} { - }1}} {net_{\chi }^{c} } net_{\chi + 1}^{c} } \right)$.

## Evaluation index and result evaluation

### Evaluation Indexes

(1) Acceptance rate

The acceptance rate represents the comparison between the number of VNRs successfully embedded and the total number of VNRs arriving at time t = 0 to time T, which is defined as formula (24):

24 $$ \mathop {lim}\limits_{T \to \infty } \sum\limits_{{{\text{t = }}0}}^{T} {{{VNR} \mathord{\left/ {\vphantom {{VNR} {\sum\limits_{{{\text{t = }}0}}^{T} {VNR_{S} } }}} \right. \kern-0pt} {\sum\limits_{{{\text{t = }}0}}^{T} {VNR_{S} } }}} $$

(2) Network average cost

The average network cost represents the resource consumption of successfully embedded VNR events, which mainly includes node resources and link embedding resources. It can be defined as the ratio of the total resources allocated to VNRs to the total number of VNRs arriving within t = 0 to T as shown in formula (25):

25 $$ Cost(G^{v} ,t){ = }\sum\limits_{{n^{v} \in N^{v} }} {cpu(n^{v} )} + \sum\limits_{{l^{v} \in L^{v} }} {bw(l^{v} ) \times hops(l^{v} )} $$

(3) Link pressure

The link pressure is a reflection of bandwidth consumption, which is expressed as the ratio of the physical bandwidth resources occupied by the successfully embedded VNR virtual link to the initial resources of the physical link, as shown in formula (26):

26 $$ LB\_Load(l^{s} ) = \sum\limits_{{v \in E^{v} ,p \in (p,v)}} {{{Map_{{l^{s} }} B(p,l^{v} )} \mathord{\left/ {\vphantom {{Map_{{l^{s} }} B(p,l^{v} )} {B(l^{s} )}}} \right. \kern-0pt} {B(l^{s} )}}} $$

(4) Network revenue

The network revenue reflects the operating income of the network provider, that is, the ratio between the profit obtained by the VNR successfully embedded and the total service time from t = 0 to T, which can be defined as formula (27):

27 $$ Revenue(G^{v} ,t){ = }\sum\limits_{{n^{v} \in N^{v} }} {cpu(n^{v} )} + \sum\limits_{{l^{v} \in L^{v} }} {bw(l^{v} )} $$

### Experimental settings and training results

**Figure 9:**
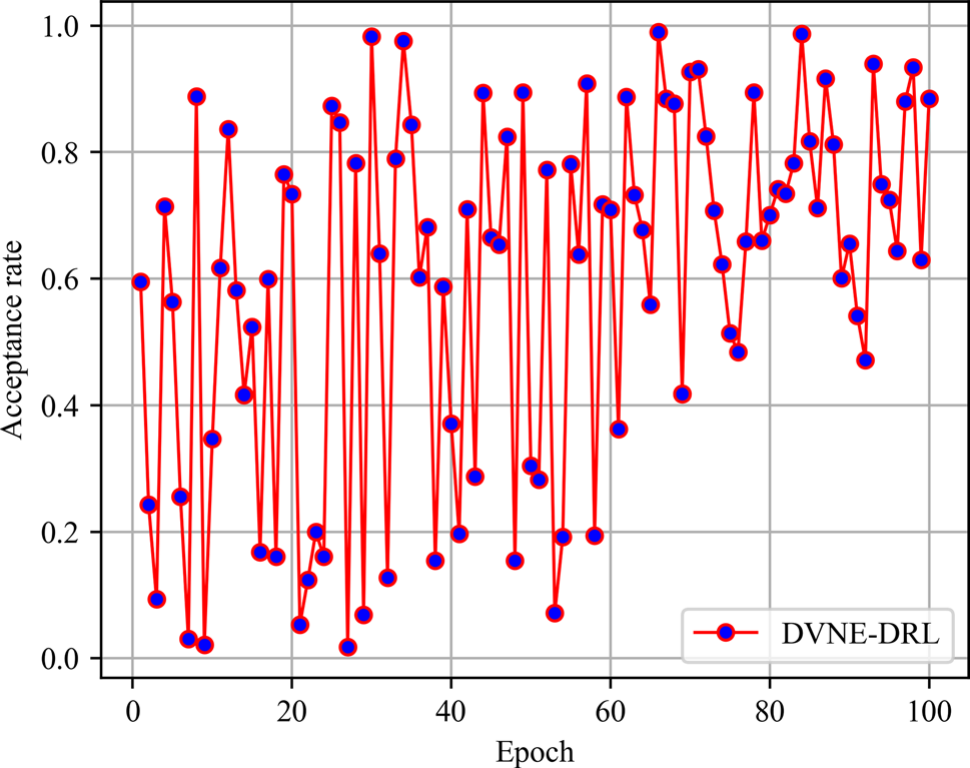
Acceptance rate.

**Figure 10:**
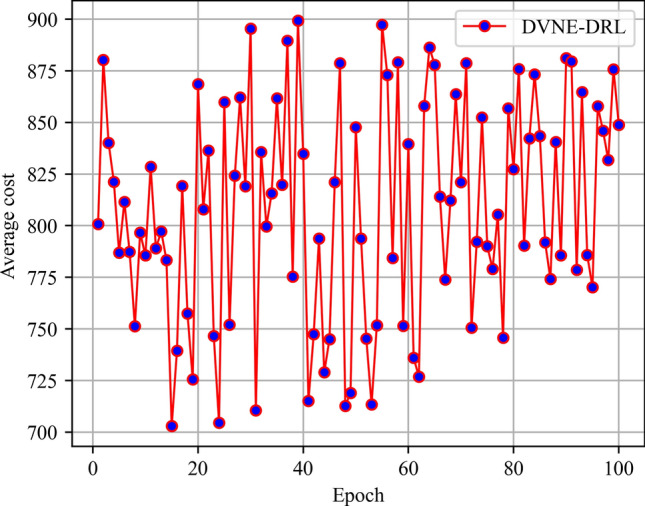
Average cost.

**Figure 11:**
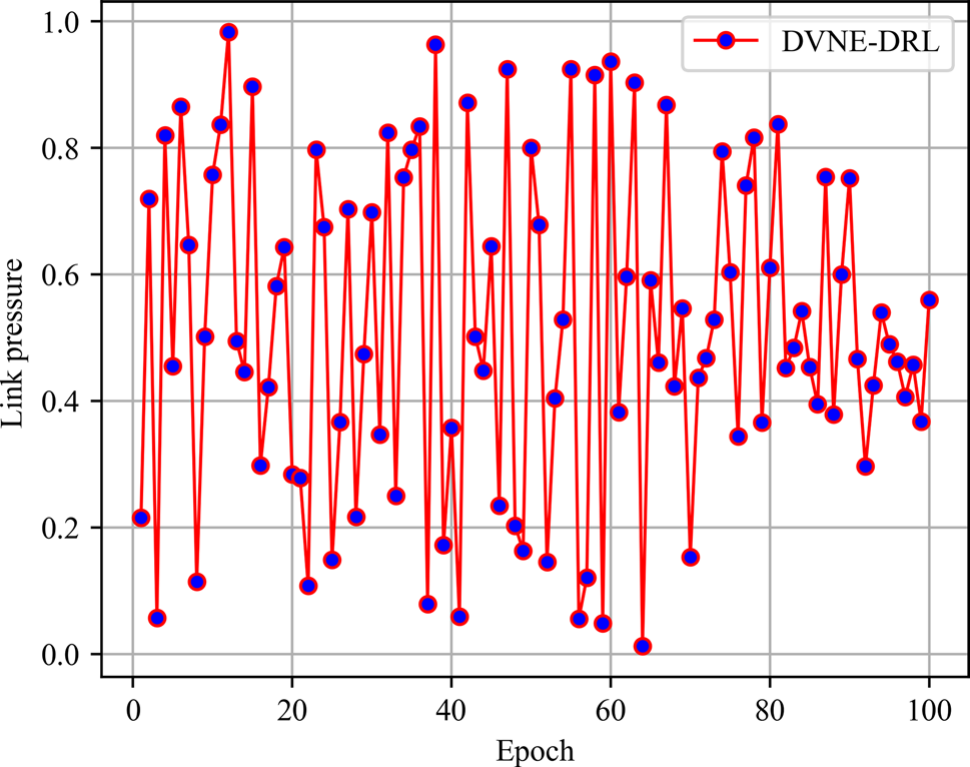
Link pressure.

**Figure 12:**
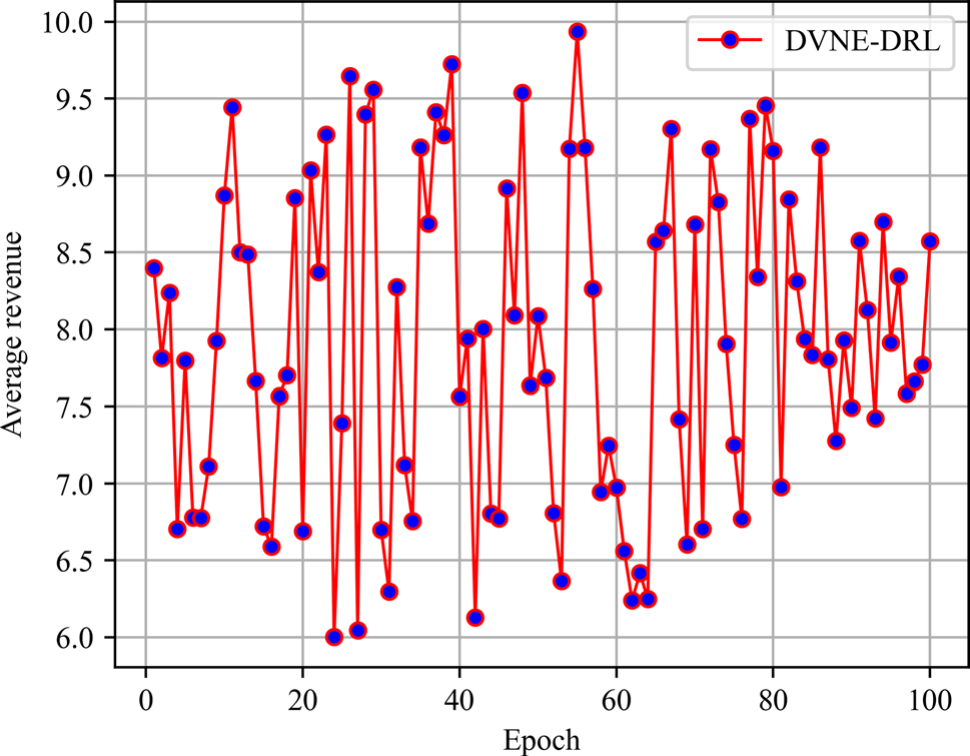
Average revenue.

**Figure 13:**
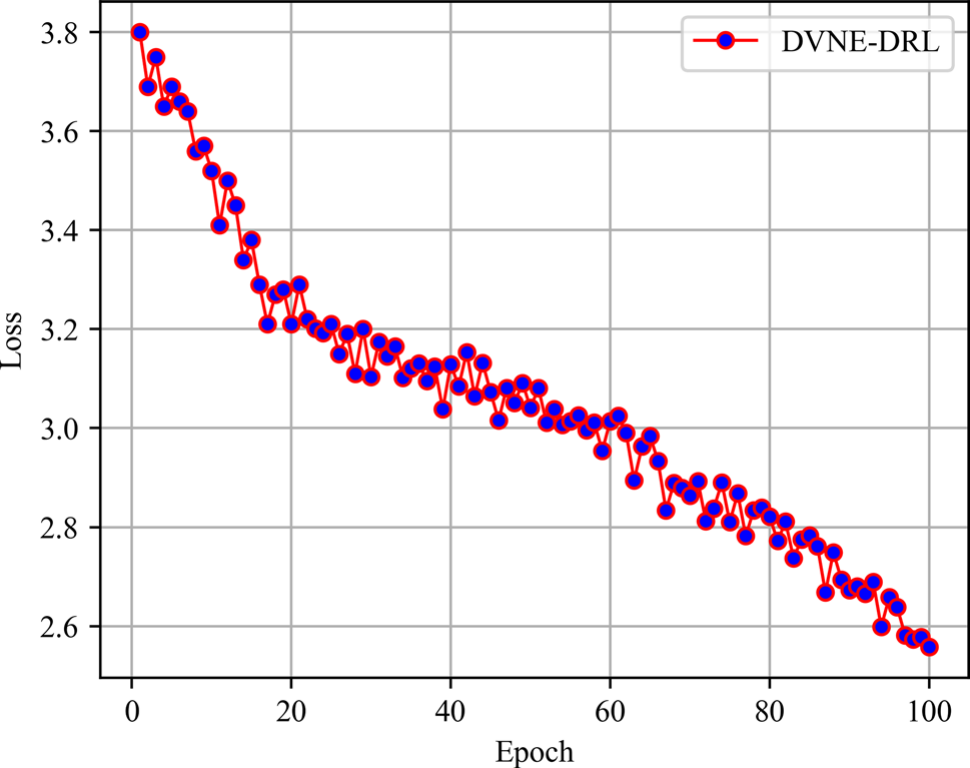
Loss on training set.

In this article, GT-ITM^34,35^ tool is used to generate the physical network and VNRs topology. Then, the feature information of the network topology is extracted and input into the reinforcement learning framework TensorFlow2.0^36,37^ built in Anaconda3 environment for simulation training and result analysis. We first build a physical network topology with 100 nodes and 800 links as an abstract form of the underlying physical network. The number of physical node resources and link resources follows the standard Poisson distribution of 50–100. Secondly, in the VNR event, the topology is the resource attribute of randomly created virtual nodes and virtual links. The number of virtual nodes is evenly distributed between 2 and 20, and the probability of connection between nodes is 0.8. The number of virtual node resource requirements and link bandwidth resource requirements is evenly distributed between 1 and 50. All node position constraints D of VNR are constant. In addition, it is assumed that the number of VNRs arriving according to the time axis follows a Poisson distribution with an average of 5, and the life of each VNR follows an exponential distribution with an average life of 500 time units. Finally, we tested the resource utilization of 1000 VNR events with different arrival time and service time within 8000 time units, and carried out the index evaluation record and analysis of the results as shown in Section "Experimental Settings and training results". Table 2 lists the specific experimental parameters, and then the DVNE-DRL agent is trained under the current network parameter environment. The test results of network embedding index and cross entropy loss are shown in Figs. 9, 10, 11, 12 and 13.

**Table 2:** Experimental environment parameter setting.

Parameters	Value
Number of physical nodes	100
Number of physical links	800
Physical node computing resource	U [50,100]
Physical link bandwidth resource	U [50,100]
Number of virtual network	1000
Number of virtual node	U [2,20]
Virtual node connection probability	0.8
Virtual node computing resource requirements	U [1,50]
Virtual link bandwidth resource requirements	U [1,50]
The lifetime of each VNR	U [100,800]
The learning rate of actor network	0.00025
The learning rate of critic network	0.0025

From the above Figs. 9, 10, 11 and 12, it can be seen that in the initial stage of DVNE-DRL training, as the parameters of the strategy network are randomly initialized, the agent will randomly take actions to explore the possibility of obtaining good results, resulting in significant fluctuations. In the middle of the training phase, as the agent obtains more historical data and embedding knowledge, they become more familiar with the network environment. Therefore, the agent will constantly search for good solutions, resulting in a larger reward signal and gradually improving stability. In the later stage of strategy network training, the agent accumulates a certain degree of reward through feedback information, and the actions taken gradually stabilize, resulting in a smaller fluctuation range of the curve.

As shown in Fig. 13, it is obvious that losses gradually decrease and become stable as the training phase progresses. Overall, the gradual stability of the testing curve during the training process proves the effectiveness of agent training, laying a good foundation for the application of DVNE-DRL agents in later testing sets.

### Result and analysis

To show the performance of DVNE-DRL proposed in this article, we selected R-ViNE^38^, D-ViNE^38^, GRC-VNE^39^ and MCST-VNE^40^ can almost cover most perspectives of existing algorithms for comparison. The description of each algorithm is shown in Table 3. We will illustrate the performance of DVNE-DRL algorithm in terms of acceptance rate, average cost, link pressure and average revenue through simulation experiments and sampling comparison chart at different time points. It is worth noting that the real-time trends of algorithm indicator data during the experimental process are displayed through line charts, while bar charts are used to record indicator data statistics for different time periods. The above two graphical methods are used to present the overall trend of experimental data indicators and the performance differences of the algorithm at different time points with emphasis respectively, in order to better demonstrate the characteristics of the algorithm. Further, Tables 4, 5, 6 and 7 extracts part of the indicator data at different times in the VNE process to show the performance of the algorithm more directly.

**Table 3:** Comparison algorithm description.

Algorithm name	Algorithm description
DVNE-DRL (our Algorithm)	This method models VNE as a markov decision process and uses reinforcement learning agents to regularly detect the state of the network environment to provide reasonable network embedding solution in real-time
MCST-VNE	This method defines a reinforcement learning-based algorithm that uses Monte-Carlo MCTS to search the action space
GRC-VNE	This method defines a new node measurement index-global resource capacity (GRC), and ranks nodes according to this measurement index
R-ViNE	This method is used to solve the linear programming relaxation of MIP corresponding to the VNE problem, incorporating a randomly determined rounding method with the goal of minimizing the cost of VNE
D-ViNE	The method based on deterministic rounding is used to obtain the linear programming relaxation of MIP corresponding to VNE problem, which aims to minimize the cost of VNE

**Table 4:** Comparison data of acceptance rate.

Algorithm name	Acceptance rate (running time)				
	2000	3500	5000	6500	8000
DVNE-DRL	0.713	0.754	0.748	0.778	0.787
MCST-VNE	0.615	0.592	0.527	0.578	0.595
GRC-VNE	0.547	0.506	0.524	0.542	0.588
D-ViNE	0.539	0.483	0.491	0.515	0.529
R-ViNE	0.435	0.382	0.384	0.389	0.417

**Table 5:** Comparison of network cost.

Algorithm name	Network cost (running time)				
	2000	3500	5000	6500	8000
DVNE-DRL	713.23	737.12	747.26	739.35	740.69
MCST-VNE	763.23	774.26	768.58	761.15	770.38
GRC-VNE	742.34	760.52	759.26	760.78	771.74
D-ViNE	779.35	789.92	785.35	799.56	815.27
R-ViNE	799.45	818.56	817.39	846.45	852.08

**Table 6:** Comparison of link pressure.

Algorithm name	Link pressure (running time)				
	2000	3500	5000	6500	8000
DVNE-DRL	0.333	0.384	0.348	0.338	0.361
MCST-VNE	0.455	0.442	0.477	0.428	0.385
GRC-VNE	0.447	0.486	0.494	0.472	0.423
D-ViNE	0.569	0.603	0.681	0.685	0.629
R-ViNE	0.625	0.621	0.707	0.759	0.791

**Table 7:** Comparison of average revenue.

Algorithm name	Network revenue (running time)				
	2000	3500	5000	6500	8000
DVNE-DRL	6.32	6.01	5.88	5.48	5.07
MCST-VNE	5.61	5.28	4.29	4.01	4.08
GRC-VNE	5.02	4.23	3.12	3.02	3.02
D-ViNE	3.12	3.01	2.76	2.15	2.28
R-ViNE	2.76	2.52	2.03	1.81	2.05

**Figure 14:**
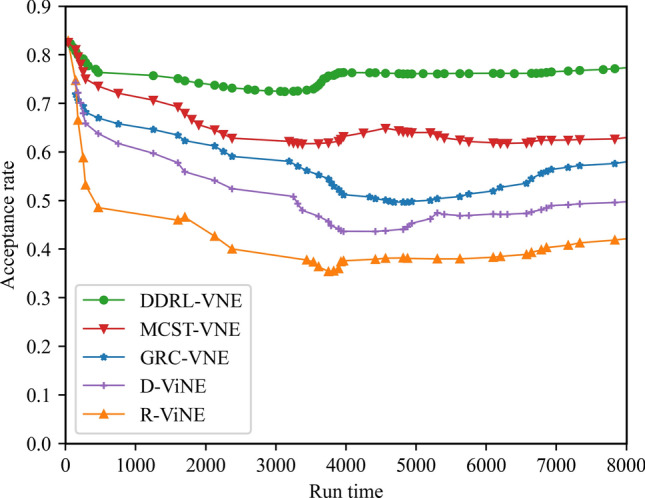
Acceptance rate (real-time comparison chart).

**Figure 15:**
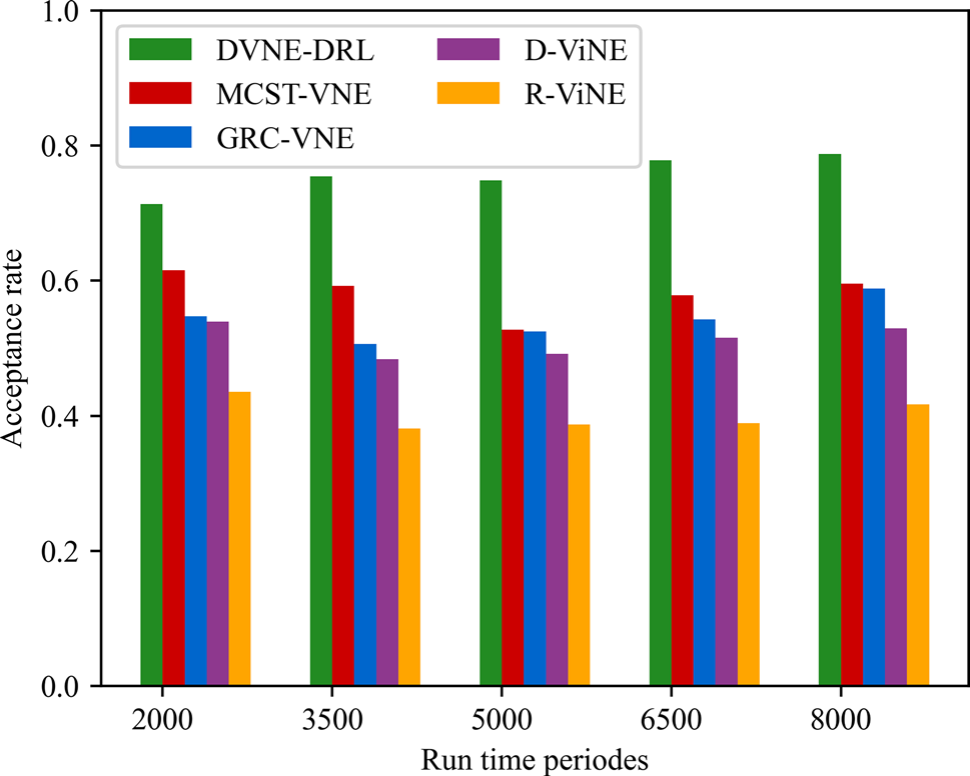
Acceptance rate (statistic bar chart).

As shown in Fig. 14 real-time comparison chart and Fig. 15 statistic bar chart of capture time period, the performance of the five algorithms is relatively stable in terms of long-term acceptance rate, because the network request type is divided into a new virtual network and a virtual network that leaves after the service ends. Therefore, the number of physical network resources will increase with the decrease of the former. As shown in Fig. 15 and Table 4, the time point samples taken between 2000 and 8000 show that MCST-VNE uses Monte Carlo MCTS to search action space, and its acceptance rate is higher than GRC-VNE, R-ViNE and D-ViNE. The acceptance rate of GRC-VNE is higher than R-ViNE, and D-ViNE due to the calculation of potential embedding capability of physical nodes. The R-ViNE algorithm is more random than the D-ViNE algorithm, and its acceptance rate is lower in the dynamic VNE environment built in this article. An important reason why DVNE-DRL is superior to the other four algorithms is that we built the dynamic process of VNE in 3.3. In the DDRL algorithm, at a specific point in time, the learning agent will monitor changes in the network environment, including resource changes and topology changes, and select a more reasonable physical node for the virtual node according to the current network conditions. This greatly reduces resource fragmentation and therefore improves VNR acceptance. At the same time, due to the interaction with the environment, the acceptance rate trend of DVNE-DRL is more stable than that of the other four algorithms.

**Figure 16:**
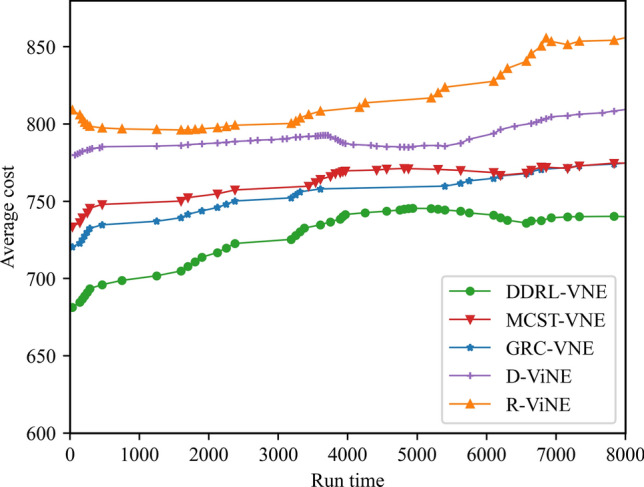
Average cost (real-time comparison chart).

**Figure 17:**
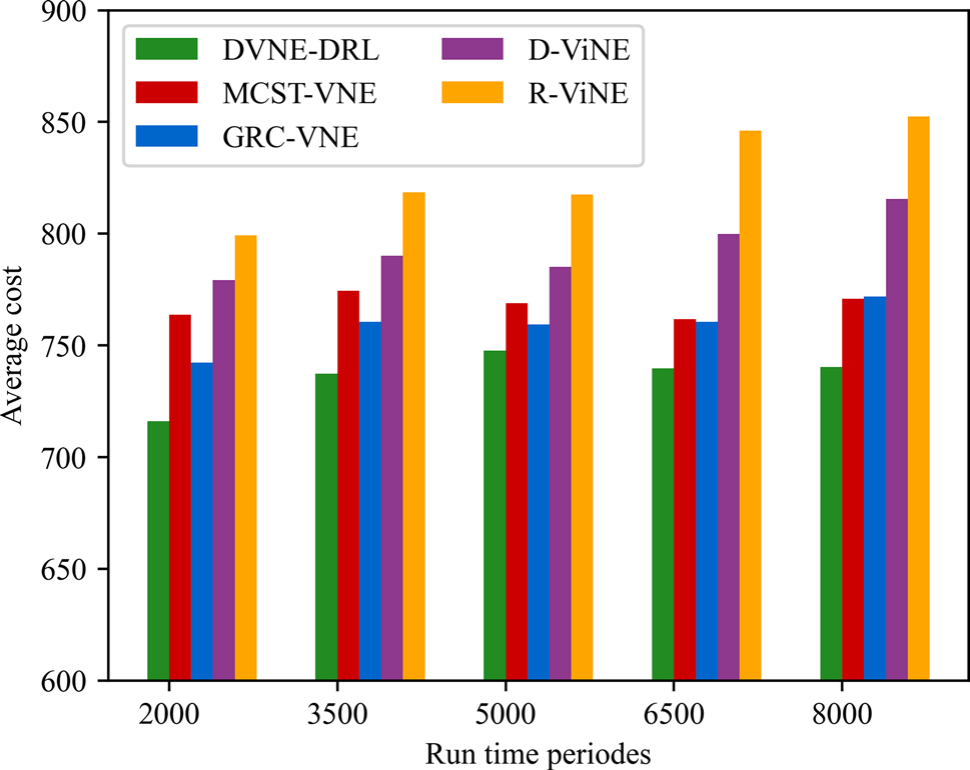
Average cost (statistic bar chart).

As shown in Fig. 16 real-time comparison chart and Fig. 17 statistic bar chart of capture time period, with the increase of the number of virtual network embeddings, the physical network resources are gradually fragmented to varying degrees, especially the link cost increases rapidly, resulting in an overall upward trend in the average cost of network embedding. As shown in Fig. 17 and Table 5, the costs of R-ViNE and D-ViNE are the highest. The overall cost of GRC-VNE is relatively stable compared with that of R-ViNE and D-ViNE, because the network topology is taken into account when calculating the embedding potential of physical nodes in advance, so the link embedding path is relatively short. MCST-VNE uses Monte Carlo MCTS to search action space, reducing link embedding costs. It is worth mentioning that, as the high acceptance rate, the overall cost of MCST-VNE is slightly higher than that of GRC-VNE, which is explained. However, none of the above four algorithms can interact with the network environment in real time. The learning agent in the DVNE-DRL algorithm will monitor the resource and topology changes in the environment in real time, select reasonable physical node embedding at different time points, and optimize the node selection scheme through the cost function to obtain a lower link embedding cost. Therefore, the DVNE-DRL algorithm proposed in this article has a lower cost at all stages. It is worth noting that the node cost is higher than other algorithms due to the high acceptance rate of DVNE-DRL, but the link embedding cost accounts for a higher proportion in the total cost calculation process. Therefore, compared with the other four algorithms, the network cost of DVNE-DRL is relatively stable.

**Figure 18:**
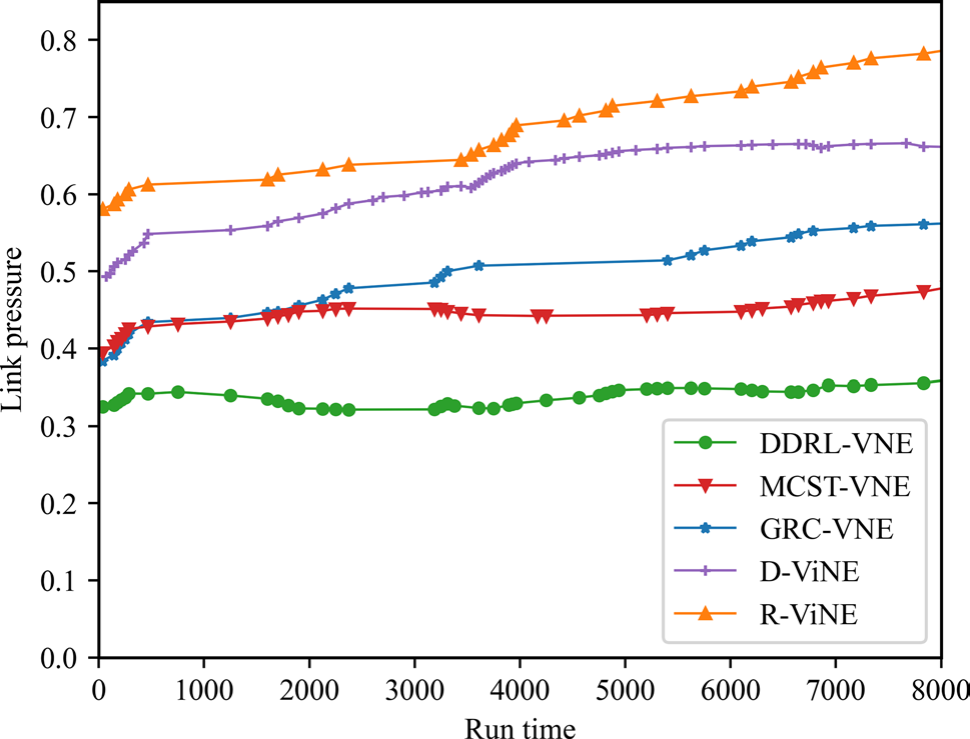
Link pressure (real-time comparison chart).

**Figure 19:**
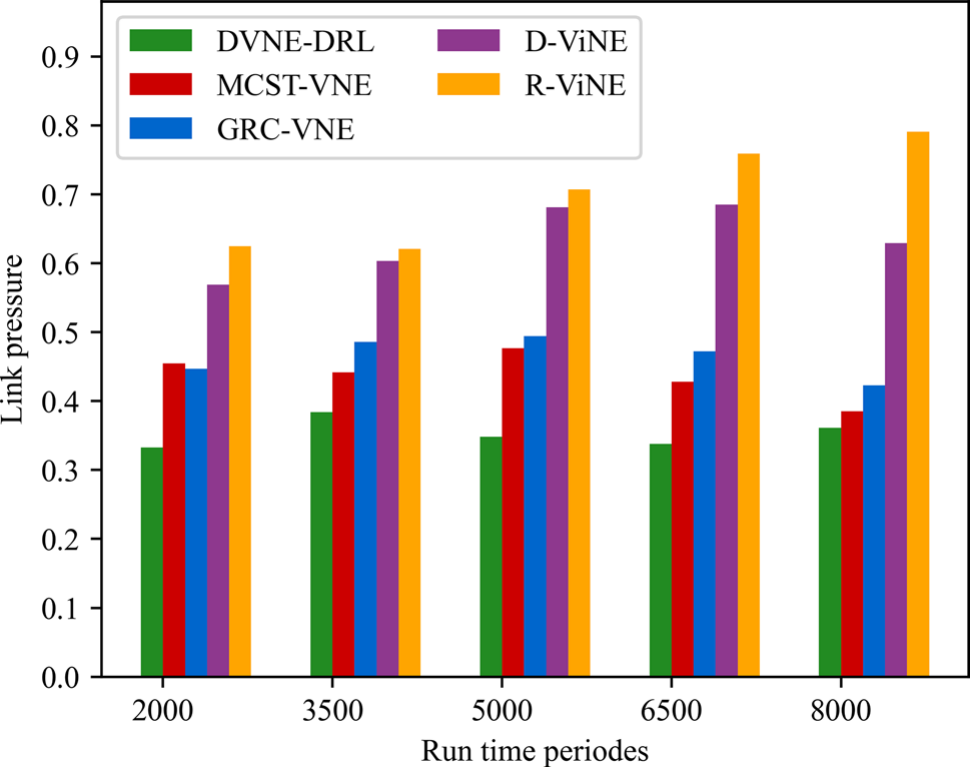
Link pressure (statistic bar chart).

As shown in Fig. 18 real-time comparison chart and Fig. 19 statistic bar chart of capture time period, the link pressure of DVNE-DRL is 15–35% lower than that of MCST-VNE, GRC-VNE, R-ViNE and D-ViNE. MCST-VNE search combines breadth first search and depth first search to find the optimal solution, which makes the link pressure relatively low and stable compared with GRC-VNE, R-ViNE and D-ViNE. Especially, when the acceptance rate is higher than GRC-VNE, the link pressure is lower, which shows the advantages of Monte Carlo tree search tree method. As shown in Fig. 19 and Table 6, the DVNE-DRL proposed in this article interacts with the dynamic network environment through learning agents, and selects nodes with similar resources and topology as the final embedding node for virtual nodes. This not only improves the embedding success rate of nodes, but also matches the corresponding physical nodes by repeatedly training the extracted network characteristics to obtain embedding nodes with shorter link embedding paths. The link embedding path usually has more hops, so choosing a shorter path will have a greater impact on the link pressure of the entire network. Therefore, compared with the DVNE-DRL of the other four algorithms, the link pressure is significantly lower, thus reducing the number of bottleneck links and improving the success acceptance rate of later embedding.

**Figure 20:**
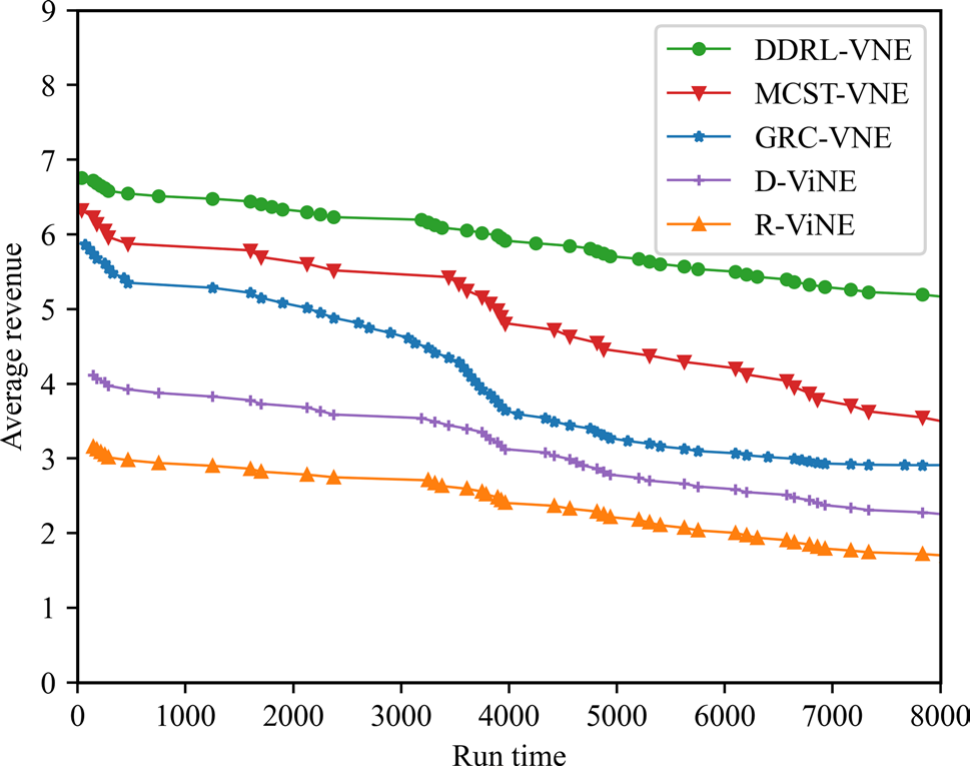
Average revenue (real-time comparison chart).

**Figure 21:**
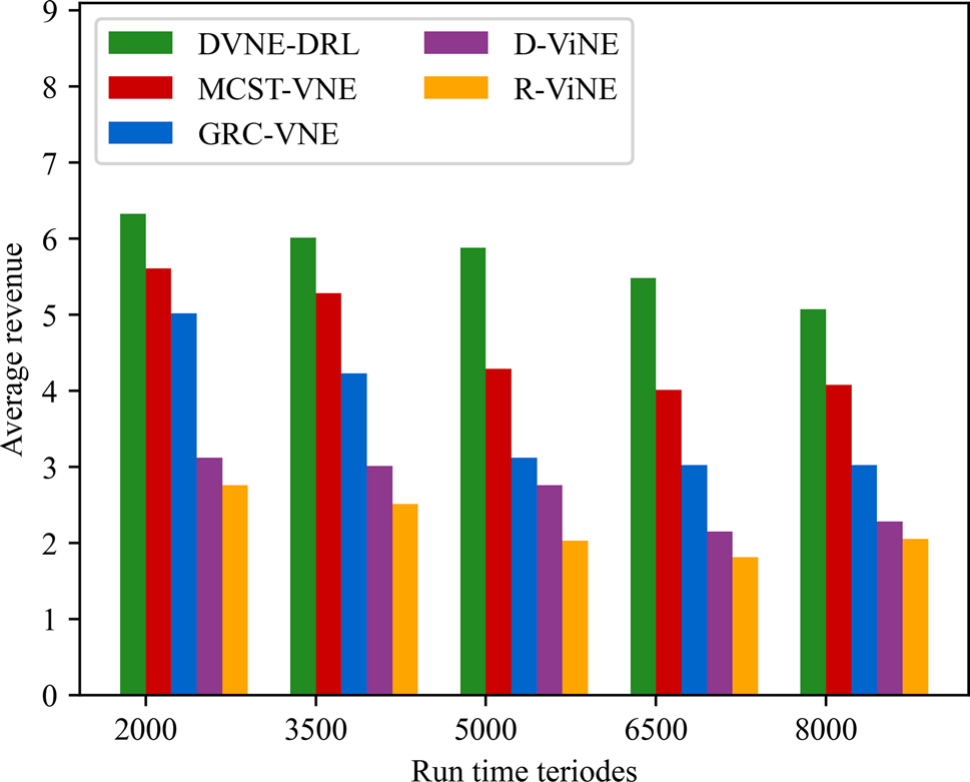
Average revenue (statistic bar chart).

As shown in Fig. 20 real-time comparison chart and Fig. 21 statistic bar chart of capture time period, DVNE-DRL has the highest network income and the gap between various algorithms is relatively obvious. This is because the network income comes from the resource cost after the VNR event is successfully embedded. That is, the more VNR events are successfully embedded, the higher the average network income will naturally be. This also leads to the comparison results of the above network revenue and the acceptance rate in Fig. 14 are generally consistent. In reality, an important goal of almost all network providers to provide services is to maximize network revenue. Therefore, an important indicator to evaluate the advantages and disadvantages of an algorithm design is whether to maximize network revenue with existing resources. As shown in Fig. 21 and Table 7, compared with traditional algorithms, DVNE-DRL can not only meet users' dynamic resource requirements, but also improve the overall network revenue, which reflects the excellent performance of the DVNE-DRL algorithm proposed in this article.

## Conclusions and future work

In view of the fact that most of the previous work ignored the dynamic nature of virtual network modeling, or could not automatically detect complex time-varying network states to provide a reasonable network embedding scheme, this article first proposed a new dynamic virtual network embedding framework, in which the user's resource requirements and the business network topology change dynamically over time to meet the actual situation of more real network resources. Then, a dynamic virtual network embedding algorithm DVNE-DRL based on deep reinforcement learning is proposed, which improves the feature extraction and matrix optimization methods of DRL and considers the characteristics of both virtual network and physical network. Finally, the performance of DVNE-DRL algorithm is verified by simulation. DVNE-DRL algorithm combines the strong understanding ability of deep learning and the decision-making ability of reinforcement learning, perceives and understands the current network information through historical data and embedded knowledge, and uses the decision-making ability of reinforcement learning to select the appropriate embedding method for the new VNR. We adopt four algorithms, MCST-VNE, GRC-VNE, D-ViNE and R-ViNE, which tend to solve the embedding/mapping problem with different strategies, and illustrate the performance of DVNE-DRL from four performance evaluation indicators: admission rate, average cost, link pressure and long-term average revenue. The simulation results show that the proposed DVNE-DRL algorithm has good performance in the evaluation index.

The future research of virtual network embedding algorithm mainly focuses on the following two aspects: (1) analyze the dynamic resource requirements of users, and refine the design of dynamic virtual network embedding framework. (2) Based on historical operational data and dynamic requirements of different dedicated services, design virtual network embedding algorithms with higher efficiency based on deep reinforcement learning to better meet the dynamic nature of virtual network embedding.

## Data Availability

All data generated or analysed during this study are included in this published article.
